# Hydrogel Dressing Containing Basic Fibroblast Growth Factor Accelerating Chronic Wound Healing in Aged Mouse Model

**DOI:** 10.3390/molecules27196361

**Published:** 2022-09-26

**Authors:** Yonghao Xiao, Hui Zhao, Xiaoyu Ma, Zongheng Gu, Xin Wu, Liang Zhao, Lin Ye, Zengguo Feng

**Affiliations:** 1 School of Materials Science and Engineering, Beijing Institute of Technology, Beijing 100081, China; 2 Department of Vascular Surgery, Beijing Luhe Hospital, Capital Medical University, Beijing 101100, China; 3 Department of Vascular Surgery, Wangjing Hospital, China Academy of Chinese Medical Sciences, Beijing 100102, China

**Keywords:** hydrogel dressing, bFGF, chronic wound healing, sustainable release, elderly subjects

## Abstract

Due to the decreasing self-repairing ability, elder people are easier to form chronic wounds and suffer from slow and difficult wound healing. It is desirable to develop a novel wound dressing that can accelerate chronic wound healing in elderly subjects to decrease the pain of patients and save medical resources. In this work, Heparin and basic fibroblast growth factor(bFGF) were dissolved in the mixing solution of 4-arm acrylated polyethylene glycol and dithiothreitol to form hydrogel dressing in vitro at room temperature without any catalysts, which is convenient and easy to handle in clinic application. In vitro re-lease test shows the bFGF could be continuously released for at least 7 days, whereas the dressing surface integrity maintained for 3 days degradation in PBS solution. Three groups of treatments including bFGF-Gel, bFGF-Sol and control without any treatment were applied on the full-thickness wound on the 22 months old mice back. The wound closure rate and histological and immunohistochemical staining all illustrated that bFGF-Gel displayed a better wound healing effect than the other two groups. Thus, as-prepared hydrogel dressing seems supe-rior to current clinical treatment and more effective in elderly subjects, which shows promising potential to be applied in the clinic.

## 1. Introduction

Skin as the largest organ and the first line of body defense, plays a vital role in resisting various viruses [1]. It is more susceptible to being damaged by accidents and senile diseases [2]. Most of the wounds will be healed in a short time. But some wounds, such as diabetic ulcers, traumatic ulcers, and infective ulcers, develop into chronic wounds that do not heal for a long time [3]. Especially with the deterioration of body function, the worldwide elderly population always suffers from slower wound healing, and is easier to form chronic wounds, which not only brings pain to the elder patient, but also consumes a lot of medical resources. There are about 5.7 million people in the USA suffering chronic wounds, and approximately 20 billion dollars is spent annually on wound management [4].

Growth factors (GFs) could regulate cell growth, differentiation, communication, and migration during the healing of different wounds. epidermal growth factor (EGF), platelet-derived growth factor (PDGF), basic fibroblast growth factor (bFGF), granulocyte-macrophage colony-stimulating factor (GMCSF), and transforming growth factor beta (TGF-β) are different GFs used topically in wound healing applications [5,6]. Naturally, the application of GFs on wound sits can significantly promote healing. bFGF involved in the physiological process of wound healing, and plays an important role in many stages of wound healing to promote mitosis of endoskin cells and fibroblasts [7,8,9]. bFGF also has strong biological activity so that it can promote wound healing in low concentrations [10,11]. Its external application on wound sites has been approved in the US, Japan, and China. Some commercial powder products containing bFGF are available in the clinic. The powders can be dissolved in saline and sprayed on the wound site. However, bFGF has a very short half-life, and is very easy to lose biological activity in the harsh environment of the wound. Hence, very frequent administering of bFGF solution must be required during the whole treatment process. This brings both economic and physiological burdens for the patient, and further occupies too many medical resources [12,13]. Consequently, it is very desirable for both patients and medical staff to prepare a local delivery system that can maintain the biological activity of bFGF and release it on the wound site sustainably.

Based on the presence of hydrophilic groups such as —NH_2_, —COOH, —OH, —CONH_2_, —CONH—, and —SO_3_H, the complex three-dimensional network formed between molecules which could maintain about 90 wt% water and 10 wt% natural or synthetic polymers, and this high-water content makes the hydrogel dressings suitable for treating dry and necrotic wounds [14,15,16]. Recently, novel wound dressings comprising different combinations of drugs, GFs, and biomaterials have been wildly studied, such as sodium alginate, hyaluronic acid, gelatin, chitosan (CS), nanocellulose, DNA, polyethylene glycol (PEG) and so on [17,18,19]. Typically, these hydrogel dressings possess a swell ratio of hundreds of percent [20,21,22]. Two kinds of drugs including an anti-bacterial drug [21,22,23] and healing-promoting drug such as bFGF [24] are usually introduced in the hydrogel dressing to promote wound healing. These hydrogel dressings achieved a good therapeutic effect in the animals. For example, Li et al. used sodium alginate/hardystonite composite hydrogel dressing without bFGF to treat nude mice with 6 weeks old and achieved a 100% closure rate after 21 days [25], whereas Liu applied silver crosslinked injectable supramolecular CS–Ag hydrogel doped with bFGF to treat diabetic SD C57BL/6 mice and achieved ~96% closure rate after 10 days [24]. The above results are inspiring, but they were obtained from a young animal model. Thus, one concerning whether the hydrogel dressings have a similar wound healing-promoting effect in the aged animal model remains, and further investigation is eagerly needed.

PEG as a typical synthetic polymer is wildly used in biomedical applications. It was reported that PEG hydrogel has good biocompatibility, is non-toxic, and low immunogenicity [26,27,28,29]. Previously, we had fabricated a PEG hydrogel containing heparin and bFGF (bFGF-Gel) by in situ Michael Addition of 4-arm acrylated polyethylene glycol (PEG) and dithiothreitol (DTT) at room temperature. The hydrogel dressings possess a suitable swelling ratio and can maintain the biological activity of bFGF with the help of heparin and sustainably release it into the wound site [30]. The in vivo test further demonstrates the as-prepared bFGF-Gel can significantly promote wound healing than the blank gel group and the group without any treatment. Although significant progresses were achieved in our previous work, some challenges remained. First, healthy and young rats were used so that it could not demonstrate the pharmaceutic effects of bFGF-Gel in chronic wounds in aged subjects since they usually have much poorer healing performances than young subjects. Second, since the current treatment in clinical is spraying bFGF solution, the clinical treatment instead of blank gel group is more convincing to be used as control group to demonstrate the application potential of bFGF-Gel in clinic.

With these in mind, the 12-month-old mice were fed for 10 months to obtain aged mice. Then, three groups of aged mice including bFGF-Gel, spraying bFGF solution (bFGF-Sol) and the group without any treatment (Blank), were used in the animal test to investigate the clinical feasibility of bFGF-Gel. The hydrogel dressing with bFGF can accelerate wound closure more than the other two groups, showing the promising potential to be applied in clinic.

## 2. Results and Discussion

### 2.1. In Vitro Evaluation of bFGF-Gel

**Figure 1:**
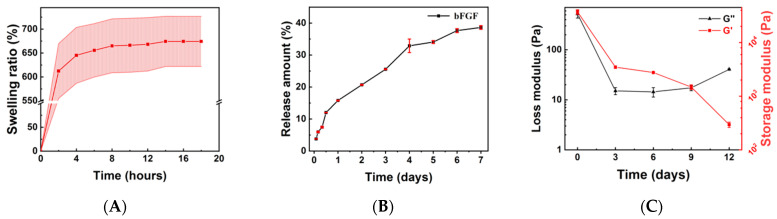
The profiles of bFGF-Gel in vitro. (**A**) The swelling behavior of bFGF-Gel. (**B**) bFGF release of the bFGF-Gel. (**C**) Storage modulus G′ and loss modulus G″ versus time during the bFGF-Gel degradation process.

Figure 1A exhibited the swelling behavior of as-prepared bFGF-Gel. The gel had a fast swelling behavior since Figure 1A showed it reached swelling balance within 2 h. Furthermore, it also possessed a large swell ratio of around 750%. The fast swelling behavior and large swelling ratio confirmed that the bFGF-Gel dressing may be able to absorb wound exudate. Figure 1B showed that bFGF in bFGF-Gel could be sustainably released for more than 7 days. At the initial 12 h, the bFGF-Gel had a large release amount, whereas the release became slower after 1 day. The release of bFGF from the gel was driven by a concentration gradient which gradually decreased with the release time. Thus, it may contribute to the slowing of the bFGF release rate [31]. After 10 days, the total release ratio reached about 38.6 ± 0.6%. The above results showed this hydrogel dressing is able to release bFGF sustainably. Figure 1C showed the modulus changes with the degradation time by rheometric measurement. Both storage and loss modulus decreased with time, implying the occurrence of degradation. The modulus decreased sharply in the first three days and slowly after 3 days. After 12 days, the storage modulus of this hydrogel is still much larger than the loss modulus.

**Figure 2:**
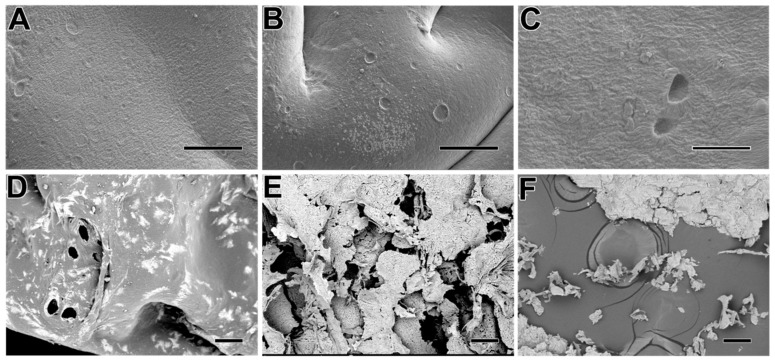
The different morphology of bFGF-Gel degraded after 0 days (**A**), 3 days (**B**), 6 days (**C**), 9 days (**D**), 12 days (**E**), 15 days (**F**) observed by SEM. The length of the scale bar is 100 μm.

The surface morphology change of the hydrogel with the degradation time is shown in Figure 2. It exhibited some holes occurring in the surface on the third and sixth day, but the surface integrity was maintained in general. However, the obvious decomposition occurred after the ninth day in Figure 2D–F. The disintegration of the hydrogel appears more pronounced from day 12 onwards, which is consistent with the results in Figure 1C. The morphology observation hinted the dressing can be used for at least three days although the modulus decreased sharply in the first three days. Consequently, combining the results of Figure 1 and Figure 2, the as-prepared hydrogel dressing can be continuously used in the wound site for 2–3 days at least as it can maintain surface integrity and possess a faster bFGF release rate within this period.

### 2.2. In Vivo Wound Repair

**Figure 3:**
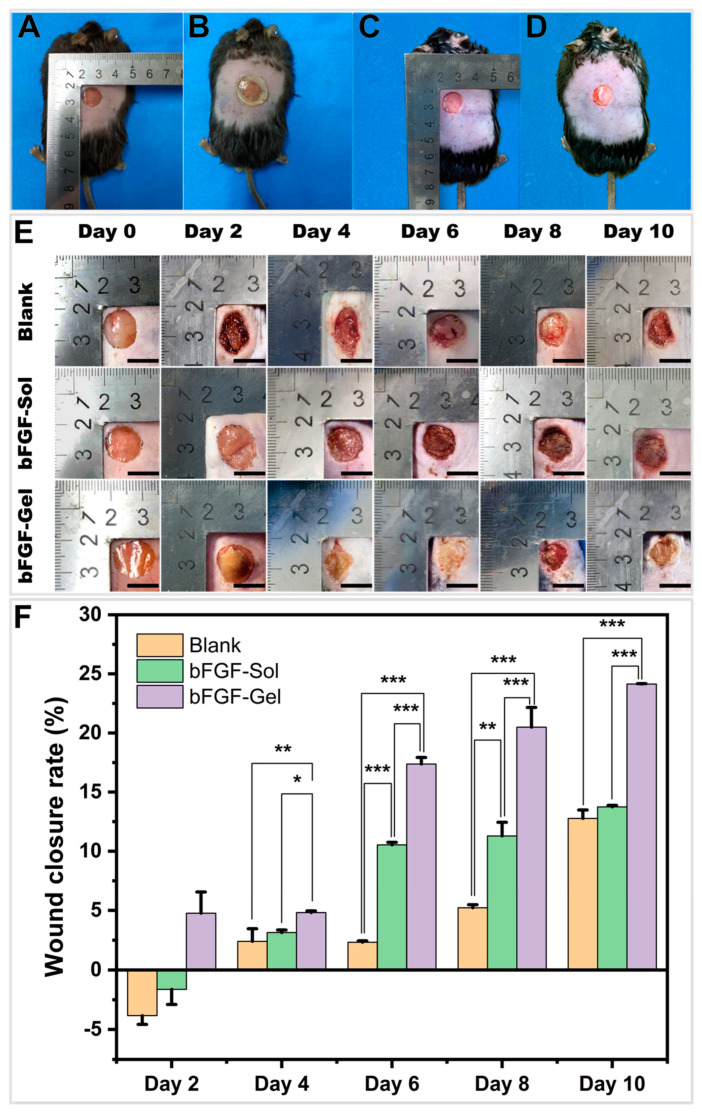
The evaluation of chronic wound healing on the aged mouse. (**A**) Full-thickness skin wound of bFGF-Gel group, (**B**) bFGF-Gel dressing covered on the wound, (**C**) Full-thickness skin wound of bFGF-Sol group, (**D**) bFGF was spraying on the wound, (**E**) Digital photos of wounds on the dorsum of mice after treatment by using no treatment (Blank), spraying bFGF-Sol and bFGF-Gel hydrogel dressings on different days after the surgery. (**F**) Summary graph of the wound closure rate of these three groups. The data were represented as mean ± SD (*n* = 3). * *p* < 0.05, ** *p* < 0.01, *** *p* < 0.001. Scale bar =10 mm.

As shown in Figure 3, after the full-thickness wound was made on the mouse’s back, the bFGF-Gel hydrogel dressing was firstly covered on the wound. Then, it was wrapped with gauze and fixed with band-aid and medical tapes. For the bFGF-Sol group, the bFGF solution was directly sprayed on the wound followed by gauze, band-aid, and medical tape treatment. While for the Blank group, the wound was directly covered by gauze, band-aid, and medical tape. The hydrogel dressing was changed once every two days, and the other two groups were also treated once every two days.

Figure 3E exhibits the gross observation of the wound healing of three groups at 0, 2, 4, 6, 8, and 10 d, whereas Figure 3F illustrated the semi-quantitative wound closure rate of three groups calculated by ImageJ. In general, the wound closure rate increases with time for these three groups. Meanwhile, significant differences were found between the three groups. Compared with bFGF-Sol and bFGF-Gel, the Blank group showed a much lower wound closure rate indicating poor healing performance. The Blank and the bFGF-Sol group did not show the starting of healing on the second day as their wound area slightly enlarged. However, the slight enlargement of the wound area on the second day might be related to the tearing of the wound during the removal of medical tape. The obvious closure for three groups was observed from the fourth day. On the sixth and eighth day, two bFGF groups exhibited a much higher closure rate than Blank group, while the bFGF-Gel group possessed the highest closure rate with statistical significance. On the 10th day, bFGF-Gel still showed a higher closure rate than the other two groups, whereas the bFGF-Sol group possessed a little higher closure rate than Blank without statistical significance. Thus, it could be concluded that bFGF-Gel achieved the best therapeutic effect and the bFGF-Sol group placed next among the three groups. However, the total closure rate of bFGF-gel in aged mice in this study seemed lower than our previous results in the young rats [13]. This difference is very likely related to the age and health of the experimental animals. On the other hand, the difference in the closure rate between the two studies also indicated a chronic wound model which fits the clinical cognition of aged patients was successfully applied in this study. Anyway, the result in Figure 3 demonstrated the bFGF-Gel dressing is still effective to promote wound healing in aged subjects. Next, since the bFGF-Sol group is the treatment currently adopted in clinic, this result further proved the bFGF-Gel dressing is superior to the current clinical treatment. Additionally, both bFGF groups showed better therapeutic effects than Blank, indicating that bFGF plays an important positive role in wound healing. The H&E, Masson, and immunohistochemical stains were carried out in the following in order to approximately clarify the role of bFGF in the four stages of wound healing including hemostasis, inflammation, proliferation, and remodeling phase [32].

### 2.3. Histology of Wound Healing Site in Mice

**Figure 4:**
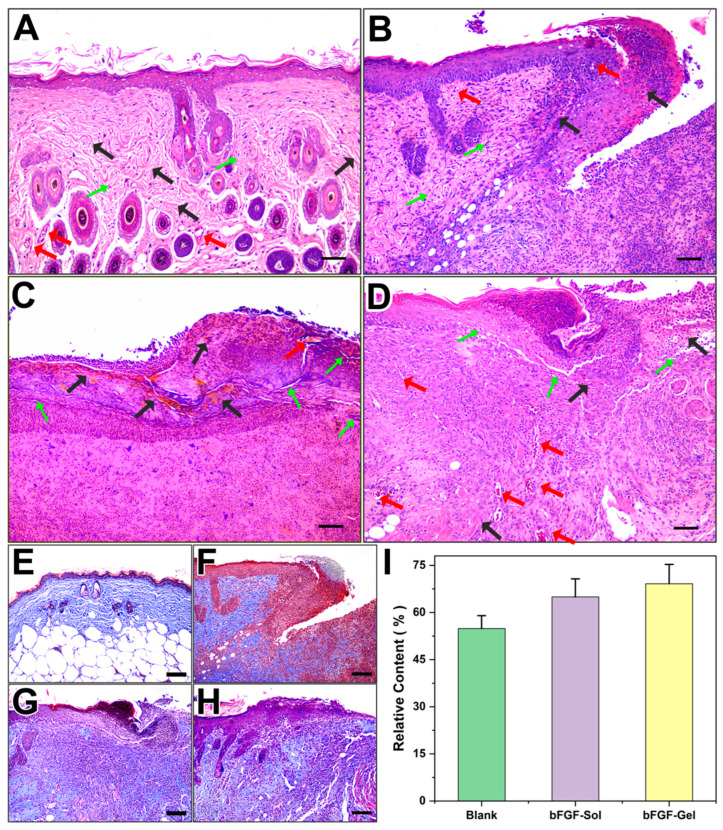
Histological analysis of wounds on the dorsum of mice by H&E staining after treatment with no bFGF, spraying bFGF, and hydrogel dressing containing bFGF for up to 10 days. (**A**) normal skin, (**B**) Blank, (**C**) bFGF-Sol, (**D**) bFGF-Gel. Scale bar =100 μm. (Green arrows: fibroblasts; black arrows: collagenous fibers; Red arrows: blood capillaries.). Masson staining images from day 10 post-operation. (**E**) normal skin, (**F**) Blank, (**G**) bFGF, (H) bFGF-Gel, (**I**) relative content of collagen in different groups compared with normal skin. Scale bar =100 μm.

To further monitor the healing progress of chronic wounds, H&E staining was conducted on the explanted skin sample on the 10th day. The normal back skin of an old mouse is shown in Figure 4A, and Figure 4B–D corresponded to the Blank, bFGF-Sol and bFGF-Gel groups, respectively. It showed the epidermis and dermis of the Blank group were not completely repaired after 10 days of self-repairing, whereas two bFGF groups repaired much better. Compared with the normal skin, Blank and bFGF-Sol groups seemed still at the early third stages (proliferation) of the wound healing process. However, bFGF-Gel group showed the best repair effect which regenerated the new skin structure similar to the normal skin. Figure 4D clearly showed the content and distribution of fibroblasts (green arrows) and collagen fibers (black arrows) of newborn skin that are superior to the other two groups. For the bFGF-Sol group, although the bFGF was directly sprayed on the wound site, the applied bFGF would lose bioactivity soon due to its short half-life time and high sensitivity. Thus, bFGF concentration on the wound site would be significantly decreased shortly after spraying. For bFGF-Gel group, in vitro test had proved that the as-prepared hydrogel can maintain the bioactivity for a long time and release bFGF sustainably so that it can maintain the bFGF concentration at a high level on the wound site. This can definitely accelerate wound healing. Additionally, the hydrogel treatment can also benefit the repair of the epidermis and dermis at the same time. This is because the wet environment provided by hydrogel can promote the migration of epidermal cells and accelerate the process of re-epithelialization of the wound, which makes the wound closed early. Consequently, the bFGF-gel exhibited obvious advantages such as maintaining exogenous bFGF concentration on the wound site and wet dressing treatment against the bFGF-Sol group (clinical treatment), so as to exhibit better wound healing performance. On the other hand, it can be also observed that the fibroblast content of the three groups is relatively low, and the fine epithelial formation is absent in most areas of the wound. It also implied the wound model in this study fits the characteristic of the chronic wound in aged species.

Collagen fiber is the most abundant protein on the skin, its regeneration also revealed the effect of the wound dressing on the wound healing process. Figure 4E–H are the results of Masson Trichrome staining. In the group of Blank, the collagen deposition was mainly distributed in the dermis upper part next to the epidermis, whereas the collagen deposition in two groups using bFGF was found in all parts of the epidermis and dermis, and the deposition was very similar to the normal skin. The semi-quantitative results of collagen content were given in Figure 4E, which showed the collagen contents of all three groups did not reach that of normal skin. It seemed bFGF-Gel possessed the highest collagen content among the three group, which was 69.1 ± 6.2% compared with normal skin. The contents of collagen in Blank and bFGF-Sol groups were 54.9 ± 4.1% and 65.0 ± 5.7% relative to the self, respectively. Although it did not show a statistical difference with the bFGF-Sol group, the collagen content of bFGF-Gel is found to be significantly higher than that of Blank in statistic. Consequently, although the presence of collagen can be found in all three groups, it seemed the bFGF-Gel possessed the best healing according to the collagen distributions and morphology in Figure 4.

**Figure 5:**
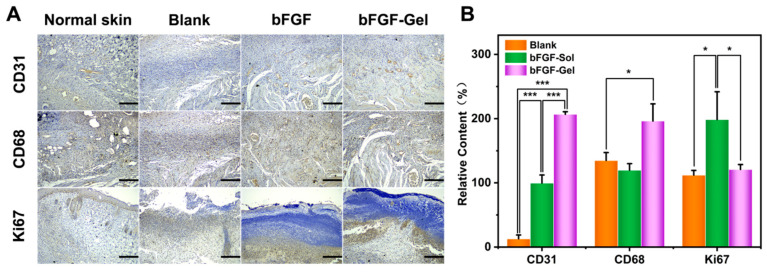
Immunohistochemical analyses of wounds on the dorsum of mice by CD31, CD68, and Ki67 staining after 10 days. (**A**) Representative images of CD31, CD68, and Ki67 staining in groups of Normal skin, Blank, bFDF, and bFGF-Gel at day 10; Scale bar = 200 μm. (**B**) Semiquantitative results of CD31, CD68, and Ki67 staining. The data were represented as mean ± SD (*n* =3). * *p* < 0.05, *** *p* < 0.001. Scale bar = 200 μm.

The blood capillary vessels of the dermis provide the necessary oxygen and nutrition to the skin. The full-thick defection of the skin also lost its capillary network. When wound healing happens, the formation of the capillaries network would correspondingly appear in the stage of inflammation. Then, with the gradual healing of the wound, the proliferation of fibroblasts further accelerates the remodeling of the extracellular matrix. Meanwhile, the number of blood capillaries continues to increase. Thus, the regeneration of blood capillaries is an important parameter to evaluate the healing process. CD31 is an important marker of endothelial cells, which can reflect the relative number of capillaries in the sample and be conducive to further understanding the repair process and repair effect of the wound surface. CD68, as the most reliable marker of macrophages, is an important reference index for the degree of the inflammatory response of wound healing. CD68 widely distributes on various macrophages including two of the most important macrophages, macrophages phenotype 1(M1) and macrophages phenotype 2(M2), involved in wound healing process. Ki67 is an essential protein in the silk division process, thus the high or low levels of Ki67 represent the amounts of cells in the proliferation phase. Figure 5A showed CD31, CD68, and Ki67 immunohistochemical staining of explanted samples on the 10th day, whereas their semi-quantitative results calculated by ImageJ were exhibited in Figure 5B. The differences in CD31 content between the three groups were quite obvious by visual observation, where the bFGF-Gel exhibited the highest CD31 content, bFGF-Sol placed next, and the Blank group was last. Their semi-quantitative results were completely consistent with visual observation, and significant differences in statistics were achieved between any two groups. It indicated that the application of exogenous bFGF is beneficial for the regeneration of blood capillaries since two bFGF groups exhibited much higher CD31 content than the Blank. As mentioned above, bFGF-Gel can sustainably release bFGF on the wound site to maintain local bFGF concentration so that it possesses the highest CD31 content. Clearly, the highest CD31 content in bFGF-Gel implied the best wound healing performance.

The differences in CD68 content in the three groups cannot be observed visually, and ImageJ was also used to calculate the average optical density of the positive region. Figure 5B illustrated there are no significant differences in statistics between Blank and bFGF-Sol group, while bFGF-Gel had a higher CD68 content than the other two groups with a significant difference in statistics. The macrophages with different phenotypes have different contributions to wound healing. M1 is pro-inflammatory and microbicidal, whereas M2 is immunomodulatory, reparative, and poorly microbicidal [33]. M1 secrete pro-inflammatory cytokines, such as TNF-a, IL-1, IL-6, IL-12, and IL-23, which possess anti-proliferative functions. In addition, M1 also will lead to tissue injury via the production of a variety of enzymes in the process of tissue reorganization. The M1 must transition to M2 after an acute inflammatory response. The balance of these two phenotypes plays a critical role in the phagocytosis of pathogens, the clearance of apoptotic cells, and the remodeling of injured tissues. Since CD68 is positive for both M1 and M2 phenotypes, the high CD68 content detected in the bFGF-Gel group may be not bad for wound healing. However, further experiments need to be done to identify M1 and M2 separately in the bFGF-Gel group in order to understand the influences of high CD68 content on wound healing.

Ki67 images showed that the bFGF-Sol group had more proliferative cells than the other two groups. As mentioned above, wound healing is divided into four stages, hemostasis, inflammation, proliferation, and remodeling. The higher proliferative cell amount in bFGF-Sol indicated this sample may be in the proliferation stage of wound healing. Since Figure 3 already showed bFGF-Gel has an obvious higher wound closure rate than bFGF-Sol, the result that bFGF-Gel had lower Ki67 content than bFGF-Sol in Figure 5B may indicate bFGF-Gel had already been in the remolding stage of wound healing. In this stage, large amounts of macrophages with the M2 phenotype would be involved. Thus, the deduction that bFGF-Gel had already been in the remodeling stage is also consistent with the result that bFGF-Gel had high CD68 content.

Summarily, immunohistochemical results showed that bFGF-Gel has better blood capillary regeneration as well as an earlier remolding stage than bFGF-Sol. Thus, it together with histological and wound closure experiments has implied that bFGF-Gel treatment may have a better therapeutic effect than current clinical treatment. Furthermore, it is noted that complete wound healing was not achieved on the 10th day. However, this experiment was performed on 22-months-old elderly mice which is equivalent to about 78-years-old human beings [34]. Thus, considering the cell aging problems, the formation of chronic wounds, and the poor self-repairing ability of the elderly subjects, the obtained results in this study are satisfactory.

## 3. Materials and Methods

### 3.1. Preparation of Hydrogel Dressing Containing bFGF

These hydrogel dressings are prepared referring to our previous work [13]. 6 mg heparin (Mn = 10,000, KehBio Co., Ltd., Beijing, China) and about 50,000 U recombinant human basic fibroblast growth factor (bFGF, Nanhai Longtime Pharmaceutical Co., Ltd., Foshan, China) was added into the solution of 20 mL PBS buffer within 1.32 g 4-Arm acrylated polyethylene glycol (PEG, JenKem Technology Co., Ltd., Beijing, China) and 0.04 g dithiothreitol (DTT, J&K Scientific Co., Ltd., Langfang, China). After forming a uniform solution, the solution was poured into a PTFE mold (10 cm × 10 cm) and then placed in an oven at 37 °C for 30 min to form the bFGF-Gel with a thickness of about 0.25 cm. After that, the bFGF-Gel was cut into round samples with a radius of 6 mm.

### 3.2. In Vitro Release of bFGF

bFGF-Gel samples were wrapped with gauze, placed in a closed Erlenmeyer flask containing 10 mL of PBS solution, and shaken in a shaker at 37 °C. At predetermined time points, the release solution was replaced by another 10 mL fresh PBS solution. The concentrations of bFGF were determined by enzyme-linked immunosorbent assay (ELISA).

### 3.3. Swelling Ratio, Degradation and Morphology

After lyophilization, the hydrogel was immersed in the PBS (pH = 7.4) buffer to investigate its swelling ratio. The mass of dry gel was recorded as W_0_. The samples were taken out at pre-determined time points. After the surface water was sucked by a filter paper, the sample mass was measured and recorded as W_t_. Thereafter, the sample was soaked in buffer again. The experiment was performed repeatedly until the sample mass did not change. The swelling ratios of these hydrogels were calculated as:

(1) $$\mathrm{swelling}\;\mathrm{ratio}=\left(W_{t}-W_{0}\right)/W_{0}\times 100\%\;$$

The degradation and morphology of the hydrogel were also investigated by putting it into PBS (pH = 7.4) buffer in a 37 °C shaker water bath. After 0, 3, 6, 9, 12, 15 days, these samples were taken out and tested by rheometer (MCR301, Anton Paar) under the condition of scanning frequency 0.1–100 Hz, strain 0.1% and 37 °C Storage modulus G′ and loss modulus G″ were recorded at 1 Hz. Besides rheological test, the surface of degraded samples was further observed by scanning electron microscopy (S4800, Hitachi) after it was sprayed with gold.

### 3.4. Wound Healing in Mice In Vivo

bFGF-Gel samples were wrapped with gauze, placed in a closed Erlenmeyer flask containing 10 mL of PBS solution, and shaken in a shaker at 37 °C. At predetermined time points, the release solution was replaced by another 10 mL fresh PBS solution. The concentrations of bFGF were determined by enzyme-linked immunosorbent assay (ELISA).

This research was approved by the Ethics Committee of Kangtai Medical Laboratory Service Hebei Co., Ltd., China. All animal care, housing, and sacrifice procedures were performed following the animal care guidelines of the Committee for Animal Research of Kangtai Medical Laboratory Service Hebei Co., Ltd., and Regulation for the Administration of Affairs Concerning Experimental Animals of China (Amended in 2017).

24 C57BL6/J (C57) mice with 12-month-old (purchased from Xinglong Experimental Animal Breeding Factory in Haidian District, Beijing) were fed at a standard feeding room for 10 months at 22 °C, and the day/night cycle was 12 h/12 h. Two mice died of natural causes, and 4 mice with far less weight were removed from the experiment. All rest elderly mice were anesthetized with xylazine (10 mg/kg) and ketamine (80 mg/kg), and then use depilatory cream (Veet, purchased from jd.com) to remove the hair on the back of the mice [34,35]. Subsequently, circular skin defects of full-thickness skin wounds of the mice were formed by a scalpel with a diameter of 10 mm. These 18 mice were randomly divided into three groups: blank group without any treatment (Blank), spraying bFGF solution (bFGF-Sol) and the experimental group (bFGF-Gel). The Blank group was directly washed off with physiological sodium chloride solution. The bFGF-Sol group was treated by spraying bFGF solution (2500 U bFGF) on the wound site at each time. For the bFGF-Gel group, the wounds were covered with the bFGF-Gel dressing (2500 U bFGF was contained in every dressing). After treatment, all wounds were covered with gauze and fixed with band-aid and medical tape. After two days, the same treatment was conducted on three groups repeatedly. The size of each wound was measured by using ImageJ. The degree of wound healing is expressed as a percentage of the original wound size. The wound closure rate was calculated using Equation (2):

(2) $$\mathrm{closure}\;\mathrm{rate}=\left(A_{0}-A_{t}\right)/A_{0}\;\times \;100\%$$

where A_0_ is the initial wound area on day zero and A_t_ is the wound area on the indicated date. The completion of wound repair was defined as a closure rate reaching 100%.

### 3.5. Histopathologic Examination

On the 10th day after the surgery, all mice were euthanized by injecting three times of narcotic. Each skin wound was softly washed three times using sterile medical saline. As well, based on the initial wound, 12 cm ophthalmic scissor was used to cut a round skin with a radius of approximately 6.5 mm for the following test. Each excised sample was fixed with paraformaldehyde for 48 h and then embedded with paraffin.

After that, the skin samples were stained with hematoxylin and eosin (H&E), Masson’s trichrome, as well as immunohistochemical stains. The primary antibodies used included anti-CD31 antibody (Abcam, ab28364), anti-CD68 antibody (Abcam, ab125212), and anti-Ki67 (Abcam, ab15580). Histological and immunohistochemical staining was observed with an inverted phase-contrast microscope at different multiples (100 ×, 400 ×, IX71, Olympus, Japan). The integral optical density values and coverage area of CD31, Ki67, and CD68 were measured by ImageJ software.

### 3.6. Statistical Analysis

All the quantitative data were obtained from at least three samples for analysis. Results were expressed as the mean ± standard deviation (SD). A one-way analysis of variance (ANOVA), followed by an LSD (least significant difference) test were used to compare three groups after different treatment methods. The Student’s *t*-test was used to compare these three different treatment groups. The difference with *p* < 0.05 was considered to be statistically significant.

## 4. Conclusions

A bFGF-Gel hydrogel dressing containing heparin and bFGF was fabricated in this study. The dressing can maintain the bioactivity of bFGF and sustainably release it into the wound site. In vivo experiment in elderly mice shows the bFGF-Gel group has the highest wound closure rate compared with the Blank and bFGF-Sol groups. The histological and immunohistochemical analysis further verified that bFGF-Gel has better wound healing performances than the other two groups. Consequently, it shows a better therapeutic effect in elderly subjects than in current clinical treatment. In conclusion, as-prepared bFGF-Gel hydrogel dressing shows the potential to be applied for chronic wound healing of elderly subjects in the clinic, which will reduce patient pain and save medical resources.

This work was aimed at investigating the application of this hydrogel dressing in older mice. However, there is an enormous demand for diabetic patients for new effective wound dressings. Therefore, this bFGF-Gel will be applied in elderly diabetic mice in our lab in the near future to further expand its applicable range in the clinic.

## Data Availability

Data are available upon appropriate requests.

## Sample Availability

Samples of the compounds are not available from the authors.
